# 

**Published:** 1891-09

**Authors:** 


*Entered at the Post Office at Austin, Texas, as Second-Class Mail Matter.*

VOL. VII. SEPTEMBER, 1891. No. 3.

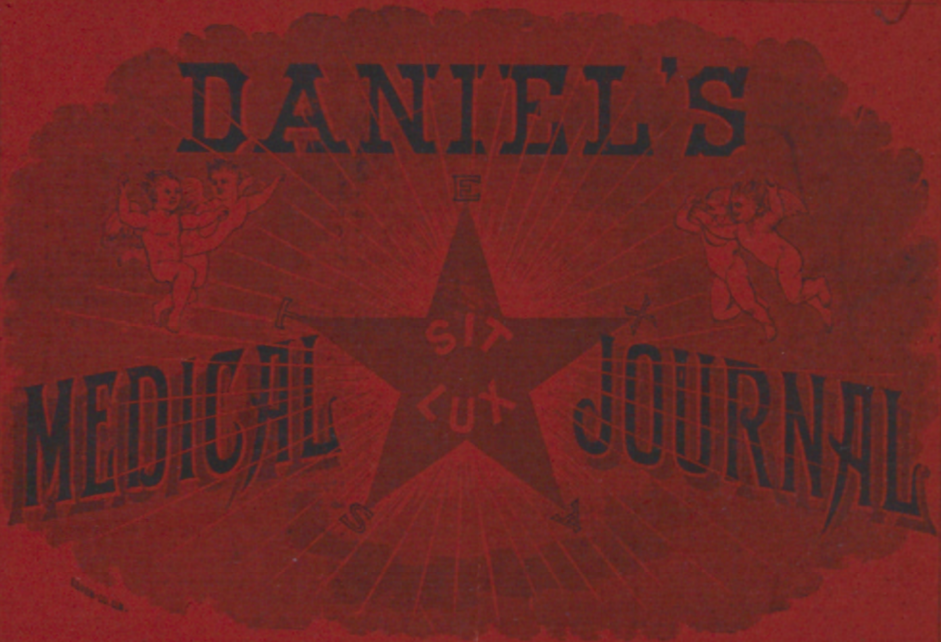

Subscription, $2.00 a Year, in Advance.— Single Copies, Twenty-fiveCents.

F. E. DANIEL, M. D., Editor and Publisher, AUSTIN, TEXAS.

